# Pulmonary Inflammatory Myofibroblastic Tumor in a Patient Treated for Pemphigus Vulgaris: A Case Report

**DOI:** 10.7759/cureus.84811

**Published:** 2025-05-26

**Authors:** Meriem Rhazari, Afaf Thouil, Sara Gartini, Hatim Kouismi, Mohamed Lakhal

**Affiliations:** 1 Department of Pulmonology, Mohammed VI University Hospital, Oujda, MAR; 2 Department of Respiratory Diseases, Faculty of Medicine and Pharmacy of Oujda, Mohammed VI University Hospital, Mohammed First University, Oujda, MAR

**Keywords:** alk-negative, benign lung tumor, inflammatory myofibroblastic tumor (imt), solitary pulmonary nodule, thoracoscopic resection

## Abstract

Inflammatory myofibroblastic tumors (IMTs) are benign tumors with diverse histological presentations. Pulmonary IMTs are particularly uncommon. Diagnosis often necessitates surgical intervention for both therapeutic and diagnostic purposes.

We report the case of a 70-year-old woman with a history of uncontrolled asthma and pemphigus vulgaris treated with rituximab and corticosteroids. She presented with chest pain and a dry cough, without systemic symptoms. Imaging revealed a solitary pulmonary nodule. Thoracoscopic resection confirmed the diagnosis of an IMT, characterized histologically by spindle cell proliferation and inflammatory infiltrates, with negative anaplastic lymphoma kinase (ALK) expression.

Pulmonary IMTs are rare and diagnostically challenging due to their overlapping features with other pulmonary lesions. Etiologies include infections, autoimmune conditions, and chromosomal abnormalities, with a potential link to IgG4 involvement in allergic and autoimmune contexts. Radiologically, they typically present as well-circumscribed solitary nodules. Definitive diagnosis often necessitates surgical excision, given the limitations of imaging and biopsy.

Pulmonary IMTs, while benign, require prompt diagnosis and management due to their potential for local recurrence and rare malignant transformation. Surgical resection remains the cornerstone of treatment, underscoring the importance of vigilant long-term follow-up.

## Introduction

Inflammatory myofibroblastic tumors (IMTs), known by several names due to the variability of their cellular composition (i.e., inflammatory pseudotumor, plasmacytic granuloma, pseudosarcomatous fibromyxoid tumor) [1], remain largely unknown and are the subject of few publications.

IMTs can affect several organs (lung, urogenital tract, etc.), although in general, IMTs are a rare type of pulmonary tumor, representing less than 1% of pulmonary masses [2]. Additionally, IMTs are often solitary and are found in individuals under the age of 40 years in more than 50% of cases [3].

A positive diagnosis of IMT is based on clinical and radiological examinations, as well as preoperative cytohistology. However, these methods cannot provide a definitive diagnosis, and thus, surgery is usually performed for both diagnostic and therapeutic purposes. Although IMTs are benign, they can be invasive and recur after excision [4]. In this work, we report a case of a pulmonary IMT in a 70-year-old woman.

## Case presentation

This case involves a 70-year-old woman who was diagnosed with asthma at the age of 26 and was on step III of the Global Initiative for Asthma (GINA) guidelines but showed poor compliance and asthma control (i.e., both daytime and nocturnal symptoms, limitation of physical activity without hospitalization in the pulmonology department or intensive care unit for exacerbation). The women also experienced the following factors related to poor asthma control: allergic rhinitis, gastroesophageal reflux, and familial atopy.

This woman is being followed up for pemphigus vulgaris and is on rituximab and systemic corticosteroid therapy for six months with gradual tapering off; she is currently on 10 mg/day. During her hospitalization in dermatology, she suffered from oppressive right chest pain associated with a dry cough with no notion of expectoration, hemoptysis, or dyspnea, occurring in the context of apyrexia, with overall preservation of her general condition. On clinical examination, the patient was found to be conscious in a room with a correct air saturation of 91%, eupneic at 24 cycles per minute, with no signs of respiratory struggle, and with slight bilateral basithoracic sibilant rales.

**Figure 1:**
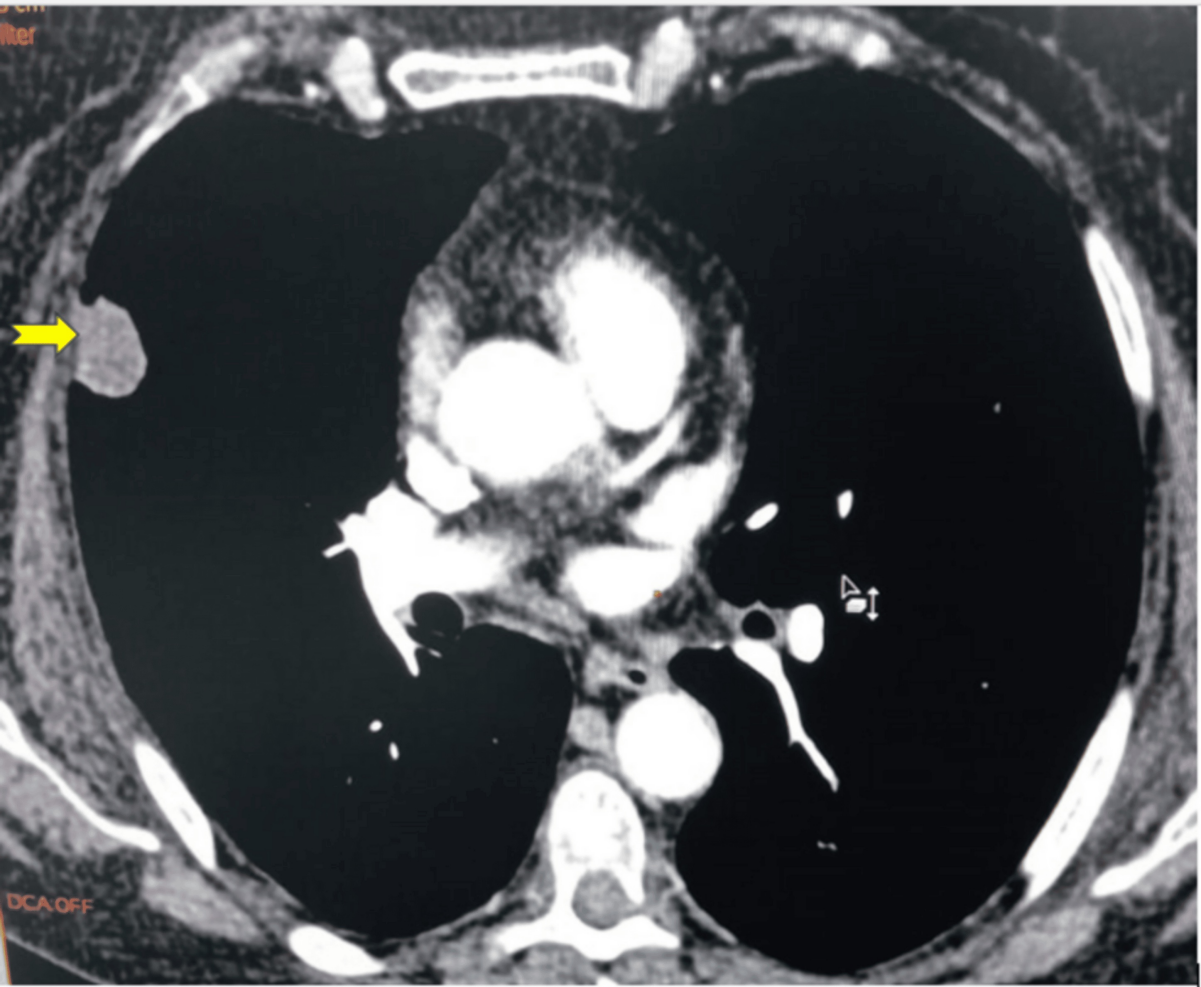
A thoracic CT scan with mediastinal window after injection of contrast medium showed the presence of a middle lobar parenchymal nodule. Yellow arrow: Middle lobar parenchymal nodule with regular contours, measuring 20 × 17 mm.

The injected thoracic CT scan showed the presence of a regularly contoured middle lobar parenchymal nodule, measuring 20 mm x 17 mm to 20 mm x 18 mm (Figure 1).

Positron emission tomography (PET) scan

**Figure 2:**
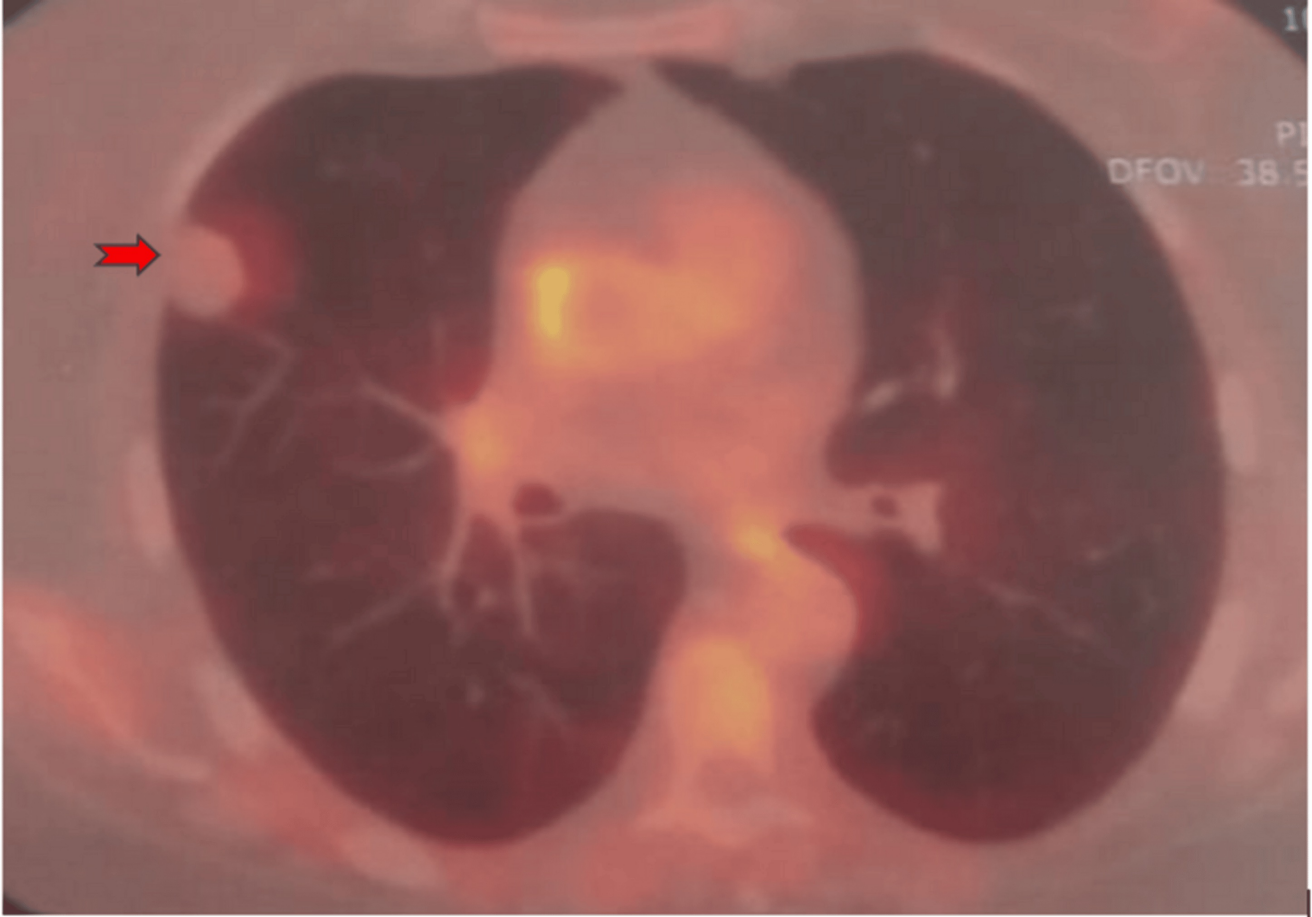
Positron emission tomography (PET) scan showing very low fluorodeoxyglucose (FDG) avidity of the 21 mm subpleural nodule in the middle lobe (red arrow), with a reference maximum standardized uptake value (SUVmax).

The 21 mm subpleural nodule in the middle lobe demonstrated very low fluorodeoxyglucose (FDG) avidity, with a reference maximum standardized uptake value (SUVmax) below background distribution function (BDF), without any clear metabolic abnormalities (Figure 2).

**Figure 3:**
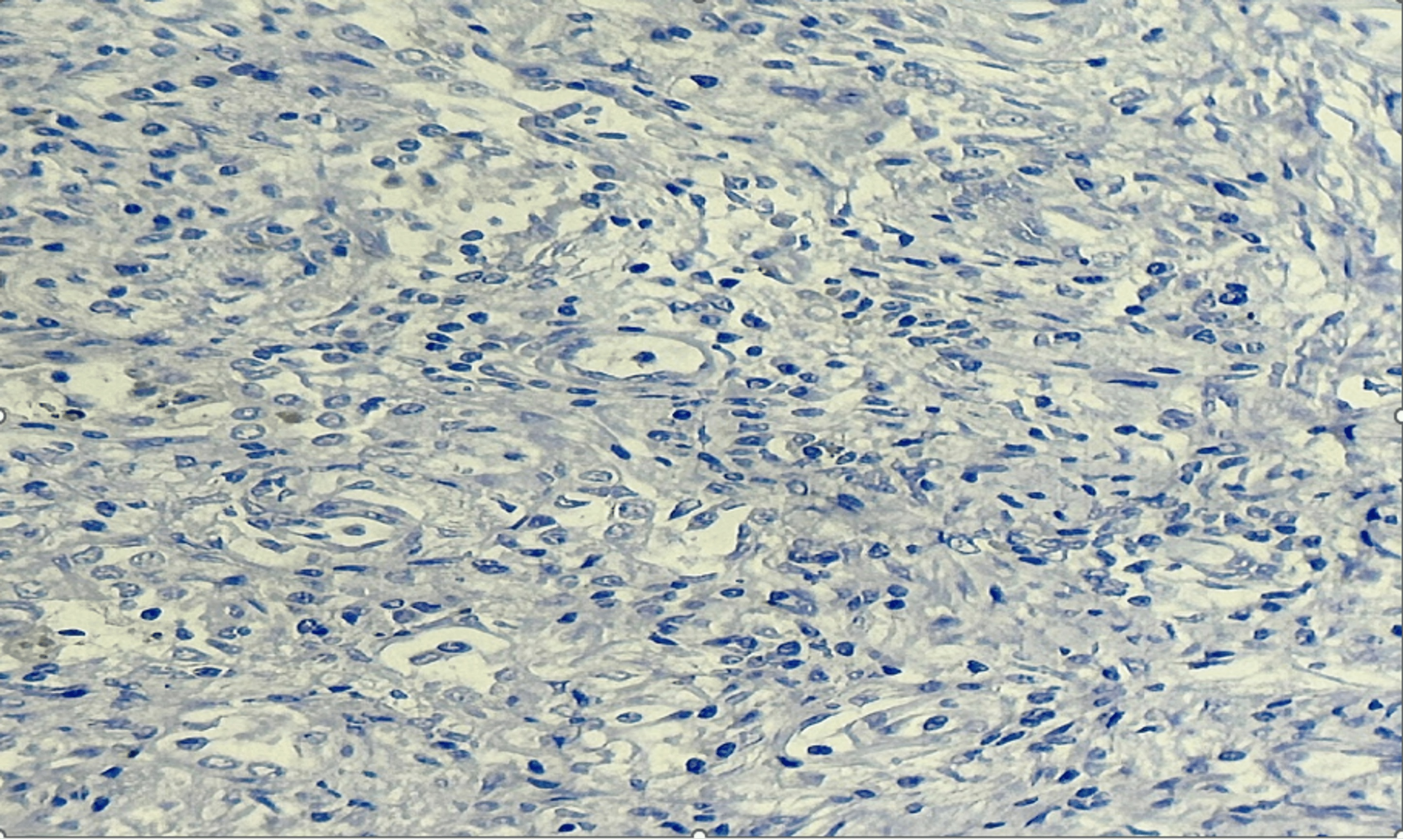
Negative staining of tumor cells with anti-anaplastic lymphoma kinase (ALK) antibody.

**Figure 4:**
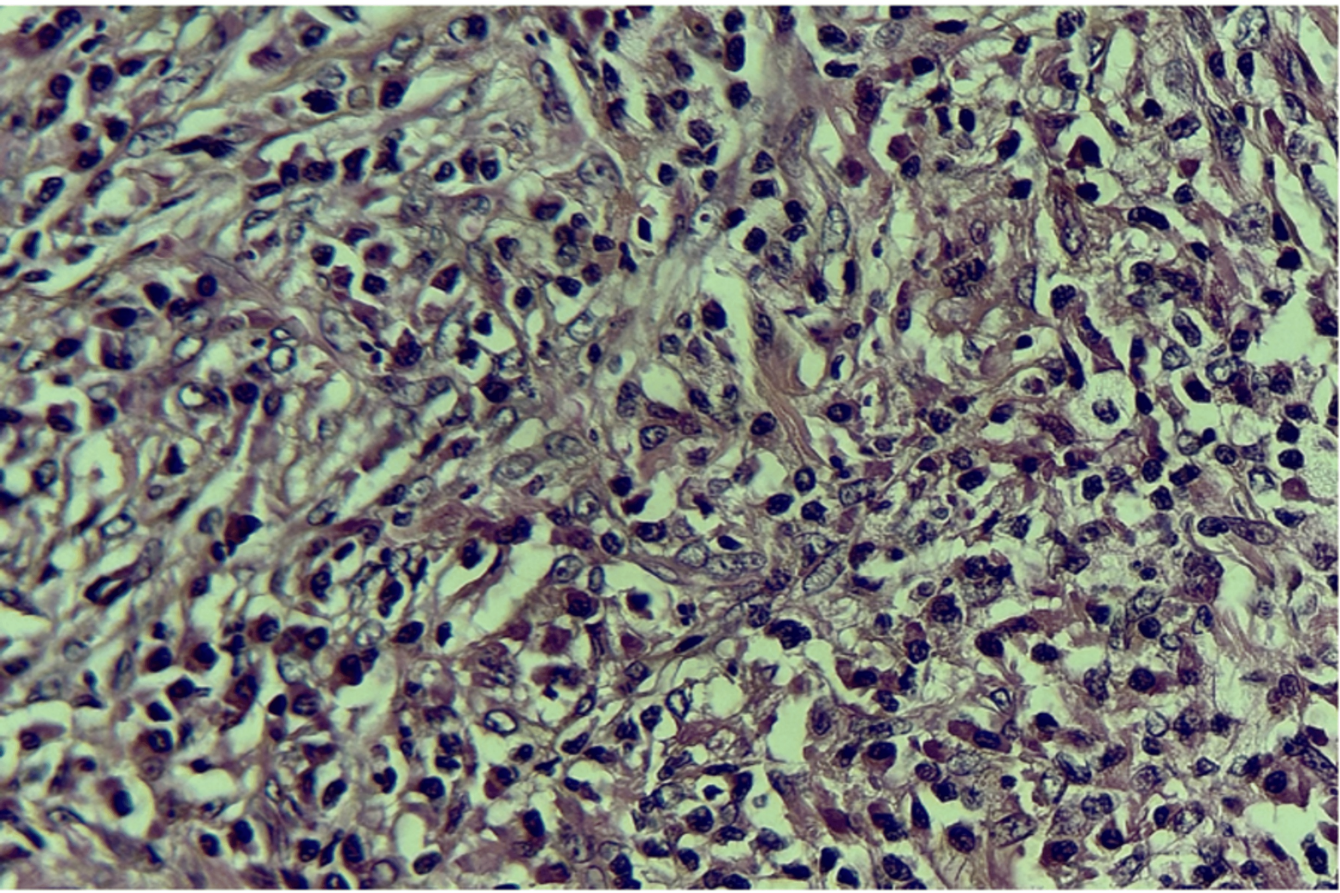
Histological image showing elongated tumor cells with moderately atypical, hyperchromatic, finely nucleated nuclei and abundant eosinophilic cytoplasm. The stroma is fibro-inflammatory, containing numerous inflammatory cells, including lymphocytes, plasma cells, and eosinophilic polymorphonuclear cells (HES ×40). HES: Hematoxylin-Eosin-Saffron

**Figure 5:**
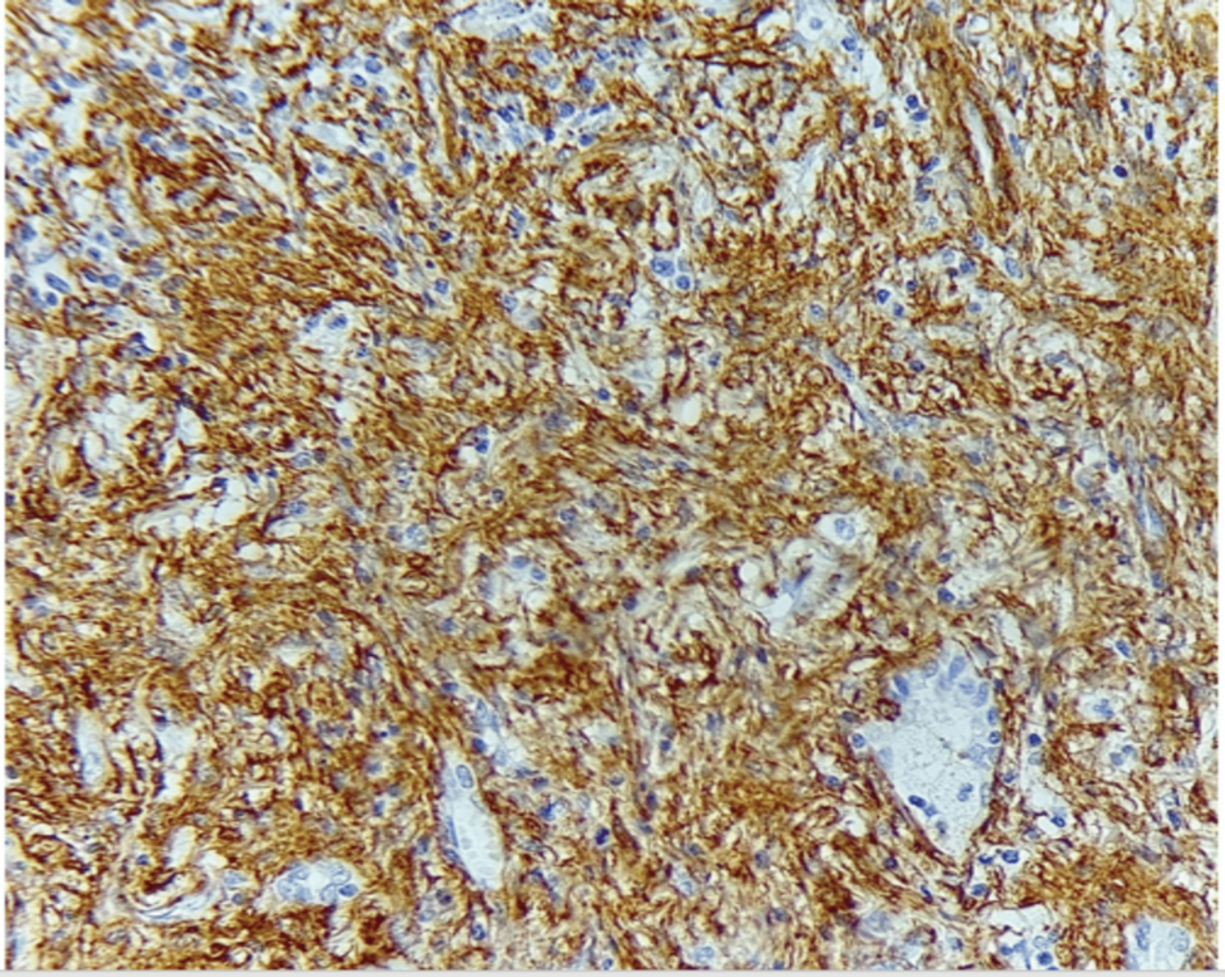
Histological image showing positive labeling of tumor cells with anti-acute myeloid leukemia (AML) antibody.

The patient underwent thoracoscopic resection of the nodule, and the histopathological study was compatible with a pulmonary IMT (Figures 3-5).

## Discussion

IMTs are a rare benign tumor with a variety of known localizations, although lung involvement is uncommon for IMTs and was identified in the first case described in the literature in 1939 [5]. IMTs are also known as plasmacytic granuloma, or inflammatory pseudotumor, depending on their predominant cellular composition [5].

In general, pulmonary IMTs are rare, representing only 0.04% to 1% of all lung tumours, and are often parenchymal and rarely endobronchial [6]. These tumors affect both genders at any age, although many studies have reported that they are most common in children and young adults [6]. Our patient was diagnosed with IMT at an advanced age.

The true incidence and prevalence of IMT are difficult to estimate, as the definition and nomenclature of fibro-inflammatory diseases are still evolving [7]. IMTs are benign tumors, but the previous literature has also reported that there are aggressive forms that invade the bronchi, mediastinum, chest wall, and diaphragm, as well as recurrent forms with a recurrence rate of 2% for pulmonary IMTs and 25% for extra-pulmonary IMTs; distant metastases occur in less than 5% of cases [2,7].

According to our research, several etiological mechanisms exist for pulmonary IMTs. Specifically, 20%-30% of cases are associated with lower respiratory tract infections, whether viral (Epstein-Barr virus or HHV8 virus), mycotic, or bacterial (*Coxiella burnetii*, *Mycoplasma pneumoniae*, *Rhodococcus equi*, mycobacteriosis) [8]. Conversely, some cases present a history of pulmonary infarction, previous radiotherapy, trauma, surgery, or an increase in IgG4-positive plasma cells [6,9]. Serum IgG4 is elevated in a limited number of conditions, such as allergic or autoimmune disorders. In bronchial asthma, IgE is involved in the response to allergens, while IgG4 is involved only late, during chronic antigenic stimulation. Therefore, it has been suggested that IgG4-producing plasma cells may act as memory cells in allergic patients. Patients with pemphigus vulgaris present with autoantibodies directed against keratinocyte desmosomes, and IgG4 is the predominant type of autoantibody detected [10]. This was the case for our patient, who had asthma and pemphigus vulgaris.

Other studies have reported evidence of chromosomal abnormalities in IMT at locus 2 of chromosome 23, leading to gene rearrangements involving the anaplastic lymphoma kinase (ALK) gene, suggesting a neoplastic rather than a reactive origin [7,9].

The circumstances of discovery of IMTs are highly variable, but they are mostly identified following patients experiencing cough, fever, chest pain, hemoptysis, and repeated infections. Additionally, in 40%-70% of cases, these lesions are discovered incidentally during a chest X-ray performed for another reason [7,8]. Our patient presented with chest pain and a dry cough.

In terms of their radiological appearance, these tumours are solitary, show well-circumscribed opacity, are 1 cm to 10 cm in diameter, and are often located in the peripheral region of the lung, especially in the lower lobes. These lesions show heterogeneous enhancement and may be associated with atelectasis and/or pleural effusions. Amorphous or dystrophic calcifications in pulmonary IMTs are more frequent in children than in adults; the presence of hilar and mediastinal adenopathies with extension of the lesion to the mediastinum has been reported in less than 7% of cases [7,11]. Our patient’s cervical-thoracic-abdominal-pelvic CT scan identified the presence of a 2 cm solitary nodule in the middle lobe.

IMTs present a challenge in terms of differential diagnosis with other entities such as granulomas, hamartochondromas, hemangiomas, solitary metastases, pulmonary sequestration, bronchogenic cysts, and hydatid cysts of the lung [8].

With IMTs, PET scans show clear uptake of FDG by the lesion and, thus, could be used to assess multi-focal forms [12]. PET was performed on our patient as part of an extension assessment and to study the hypermetabolic nature of the lesion.

Radiographic images, bronchoscopy, and percutaneous fine-needle biopsy are considered insufficient for IMT diagnosis, and thus, surgery is essential for their diagnosis and treatment [13]. This was the case for our patient, who underwent thoracoscopic resection of the nodule.

According to the World Health Organization’s (WHO) histological classification of lung tumours, IMTs belong to the mesenchymal tumours and are composed of a proliferation of myofibroblast and fibroblast spindle cells, a variable inflammatory infiltrate of plasma cells, lymphocytes, histiocytes, eosinophilic polynuclear cells, and a fibrous connective component. The borderline between malignancy and benignity is not yet clearly defined for IMTs, as these tumors can sometimes present aggressive histological criteria such as foci of necrosis, cyto-nuclear atypia, frequent mitosis, high cellularity, and vascular invasion. The distinction between malignancy and benignity in IMTs remains unclear, as these tumors can occasionally exhibit aggressive histological features, which require regular surveillance due to the possibility of secondary malignant evolution [9].

In terms of treatment for IMTs, surgical resection must be as complete as possible, including all the invaded anatomical structures. Such surgery may involve segmentectomy, lobectomy, or even pneumonectomy due to the invasive nature of the lesion [6,7] and to avoid local recurrence, which may be sarcomatous in nature [6]. However, if surgery is contraindicated, corticosteroid therapy may be used, as well as radiotherapy, antibiotic therapy, or chemotherapy, which have been tried for multiple, recurrent, or non-operable IMT forms with extensive mediastinal invasion. Spontaneous regression of IMT has also been reported [13].

Finally, the evolution of IMTs is usually satisfactory, although there is a risk of local recurrence and malignant transformation. Post-operative monitoring is indicated with an interval of up to 11 years [14].

## Conclusions

IMTs are rare, benign tumors with varied histological presentations. Pulmonary IMTs are particularly rare and difficult to diagnose. The diagnosis of pulmonary IMTs is challenging due to their resemblance to other pulmonary lesions. A definitive diagnosis often requires surgical excision, as imaging and biopsy have limitations. Although pulmonary IMTs are benign, they can recur locally and, in rare cases, transform into malignancy. Prompt diagnosis and surgical resection are essential, and long-term follow-up is necessary for monitoring.
